# Review on the Management of Primary Congenital Glaucoma

**DOI:** 10.5005/jp-journals-10008-1192

**Published:** 2016-02-02

**Authors:** Julia Yan Yu Chan, Bonnie NK Choy, Alex LK Ng, Jennifer WH Shum

**Affiliations:** Clinical Assistant Professor, Department of Ophthalmology, The University of Hong Kong Hong Kong; Clinical Assistant Professor, Department of Ophthalmology, The University of Hong Kong Hong Kong; Clinical Assistant Professor, Department of Ophthalmology, The University of Hong Kong Hong Kong; Clinical Assistant Professor, Department of Ophthalmology, The University of Hong Kong Hong Kong

**Keywords:** Primary congenital glaucoma, Childhood glaucoma, Goniotomy, Trabeculotomy, Pediatric glaucoma surgery.

## Abstract

Despite being documented in medical history from over 2400 years ago, primary congenital glaucoma (PCG), being a disease with low incidence rate, remains a challenge to ophthalmologists.

The article provides a broad overview on the pathophysiology and diagnostic approach to PCG with major emphasis on the treatment options of PCG. While reviewing on the well-established treatment options, namely goniotomy, trabeculo-tomy and combined trabeculotomy-trabeculectomy, emphasis has also been made to recent updates on secondary treatments: trabeculectomy, antimetabolites, glaucoma-drainage devices and cyclodestructive procedures.

It is, however, important to note that the rarity of PCG places limitations on study design, most studies are, thus, retrospective, nonrandomized and have different definitions of surgical success. Ophthalmologists need to interpret the results with critical thinking and formulate individual treatment plans for each patient.

**How to cite this article:** Yu Chan JY, Choy BNK, Alex LK Ng, Shum JWH. Review on the Management of Primary Congenital Glaucoma. J Curr Glaucoma Pract 2015;9(3):92-99.

## INTRODUCTION

Primary congenital glaucoma (PCG) is rare disease which constitutes a diagnostic and therapeutic challenge to ophthalmologists. The nature of the disease and the challenge it poses both contribute to its potentially devastating impact on a child’s vision. In this article, we provide an overview and update on the presentation and treatment options of PCG.

## HISTORICAL BACKGROUND

The first documentation of PCG can be dated back to 400 BC, when hippocrates recognized abnormal enlargement of the eyes in infants. It was not until early 18th century when Berger first linked the elevated intraocular pressure to the enlargement of the globe. In 1869, von Muralt established the classical form of buphthalmos, enlargement of the eyeball, as a form of glaucoma.

Subsequent anatomical dissections performed in the late 1800s and early 1900s pointed toward the malformed angle structure as culprit of PCG. The Manual of Human and Comparative Histology published in 1873 described the angle drainage system, including anatomical structure of Schlemm’s canal and Descemet’s membrane, referring to them as an anterior lymphatic drainage system.^1^ The Atlas of the Pathological Anatomy of the Eyeball, translated to English from German by William Gowers in 1875, established that angle structure malformation as the culprit for PCG.^2^ The introduction of goniotomy shortly followed in 1938, introduced by Barkan.

## CLASSIFICATION

According to the latest consensus by the World Glaucoma Association, childhood glaucoma is classified as primary or secondary. Primary congenital glaucoma, together with juvenile open-angle glaucoma, constitutes the primary childhood glaucoma. Primary congenital glaucoma can be further subcategorized by its age of onset. Primary congenital glaucoma with onset at birth to less than 1 month old is referred as the neonatal/ newborn onset PCG while late onset PCG is defined as PCG with its onset after 2 years of age. Children suffering from infantile onset PCG would have an onset between neonatal/newborn and late onset glaucoma (i.e. >1-24 months old).^3^

The Hoskins classification identifies the area of dysgenesis (Table 1). Primary congenital glaucoma refers to glaucoma due to isolated trabeculodysgenesis. The angle is maldeveloped, with absence of angle recess and iris often inserted directly onto the trabecular meshwork.

**Table 1:** **Table 1:** Hoskins classification

*Trabeculodysgenesis* *Iridotrabeculodysgenesis* • Stromal defects: hypoplasia, hyperplasia • Anomalous iris vessels • Structural defects: coloboma, aniridia *Corneotrabeculodysgenesis* • Anomalies, such as Axenfeld’s, Rieger’s and Peters	

## EPIDEMIOLOGY OF PRIMARY CONGENITAL GLAUCOMA

The incidence of PCG varies across countries and ethnic group. The incidence in the western countries, such as Ireland, Britain and the USA, lies within 1:10,000 to 1:70,000.^4-8^ In Saudi Arabia, Southern India, and among the Gypsy population in Slovakia, the incidence of PCG is significantly higher, quoted between 1:1,250 and 1:3300.^4,5,9,10^ This is thought to be due to the higher incidence of consanguinity. The relationship between consanguinity and higher incidence of PCG is further supported by the statistically significantly higher rate of consanguinity in the parents of PCG than that of the parents of secondary congenital glaucoma patients.^8^ In China, PCG constitutes 5.1% of all congenital ocular disorders.^11^

The mean age of presentation of PCG occurs at an earlier age in races with higher incidence. It ranges from 3 to 4 months among Asians, Saudi Arabians and Indians to 11 months in Western countries.^8,9,12^ While the incidence and age varies, a slight male predominance is evident in both Asian and Western countries, around 65%.^8,9,12^

## GENETIC ASPECT

Most PCG cases occur sporadically. They are familial in 10 to 40% of cases, usually with autosomal recessive inheritance and variable penetrance.^5,13^

The CYP1B1 gene is an autosomal recessive gene located at chromosome 2p21, and is a culprit gene for PCG. The mutation exhibits variable expressivity, but demonstrates almost complete penetrance. The gene mutation is prevalent in Asian PCG patients, presenting in 70% of Iran patients,^14^ 74% of South Korean patients,^15^ but surprisingly only 20% of Japanese patients.^16^ The CYP1B1 mutations have also been documented to be present in patients with juvenile open angle glaucoma in Korea and Iran, indicating a possible etiological link between PCG and juvenile open angle glaucoma.^14,15^

## PRESENTATION

Primary congenital glaucoma commonly presents bilaterally, and this is observed across Western and Asian countries alike.^8,9^ The classical triad of symptoms in PCG is epiphora, photophobia and blepharospasm, but could be absent in rare occasions.^17^ In fact, photophobia and blepharospasm is found in only 7.5% of patients at first presentation, while epiphora is only present in 3.3%.^8^ Cloudy cornea and buphthalmos account for the most common presenting sign, found in over 40% of patients. Other presenting symptoms and signs including tearing,^18^ lack of eye contact, facial birthmarks, pupillary abnormalities and nystagmus, but these only account of less than 2.3% of symptoms at first presentation.^8^

## CLINICAL APPROACH TO PRIMARY CONGENITAL GLAUCOMA

While early diagnosis is crucial, this may be difficult in early stages, which is not made easier by a lack of marked asymmetry. A high index of suspicion is key, followed by detailed history taking and clinical examination, with examination under anesthesia (EUA).

### Clinical Examination

The examination includes checking pupillary reflex, refraction, axial length and keratometry. The quality of the cornea should be examined and presence of corneal edema, scarring and Haab’s striae should be documented, preferably with photo or drawing. The horizontal and vertical corneal diameter should be measured. In newborns, the normal horizontal diameter is 9.5 to 10.5 mm and that in a 1-year-old is 10 to 11.5 mm. Horizontal corneal diameter of over 12 mm in a newborn should prompt suspicion.

### Intraocular Pressure

The intraocular pressure (IOP) at first presentation and subsequent follow-up should be charted. It is important to note that anesthetic agents are well known to influence the IOP (Table 2), affecting the accuracy of IOP documentation during EUA. Most general anesthetics and central nervous system (CNS) depressants decreases the IOP, except for ketamine, which increases the muscle tone of the extraocular muscles, paradoxically increasing the IOP. Succinylcholine is also known to cause a significant transient increase in IOP, with its peak at 20 to 30 seconds post injection. The underlying mechanism could also be related to its effect on the extraocular muscle, since succinylcholine has been documented to cause extraocular muscle contraction.^19,20^ Chloral hydrate has the least effect on IOP, followed by ketamine.

### Gonioscopy

The angle of healthy infants differs from that of healthy adults, infants have a less distinct Schwalbe’s line, less pigmented trabecular meshwork, and a translucent uveal meshwork, making the junction between scleral spur and ciliary body less clear. The angle in PCG patients differs from that of healthy infants. The iris inserts anteriorly to the trabecular meshwork, resulting in a flat or a concave insertion. Anomalous iris vessels may also be seen as loops branching from the major arterial circle. The peripheral iris may be covered by fine fluffy tissue.^25-27^

**Table 2:** **Table 2:** Medications/agents that influence the IOP

		*Agent decreasing* * IOP*		*Agents increasing* * IOP*	
Induction agents		Nitrous oxide		―	
		Cyclopropane			
		Halothane^21^			
Muscle relaxants		Tubocurarine^21^		Succinylcholine^22^	
		Gallamine			
General		Barbiturate		Ketamine^23^	
anesthetics		Morphine			
		Sevoflurane^24^			

### Fundoscopy

Similar to the adult onset open angle glaucoma, the cup disk ratio (CDR) is increased in PCG patients. There are, however, several important differences. In adult onset glaucoma, rim thinning preferentially occurs at the inferior and superior rim due to the abundance of nerve fiber layer in that area.^28^ In PCG, however, the glaucomatous cup enlarges circumferentially, as the scleral canal is uniformly stretched in all directions.^29^ Moreover, reversal of the cup may be possible upon normalization of IOP, due to the high resilience of optic nerve head connective tissue and the elasticity of the lamina cribrosa during infant development.^30^ However, reversal of cupping is not universal, as studies have observed continued thinning of the optic nerve head and retinal nerve fiber layer thinning despite IOP lowering.^31^

## TREATMENT

### Medical Management

Due to significant anatomical anomaly of the anterior drainage angle, medical treatment has a limited role in controlling the IOP in PCG, reducing IOP effectively in less than 10% of PCG patients.^32^

Table 3 below highlights different classes of anti-glaucoma medication and their effects in controlling intraocular pressure in PCG patients.^33-35^

The unfavorable efficacy, high nonresponder rate as well as a lack of long-term safety profile all contribute to the limited use of medical treatment in treatment of PCG. Most parents also find administration of compliance of eye drops in children difficult. The role of medical treatment in PCG is, therefore, generally supportive, with the aim of reducing the cornea opacity before surgery, as an interim treatment while waiting for surgery, or as an adjunct to maximize IOP reduction postoperation.

### Surgical Treatment

The major surgical options for PCG include angle surgery, drainage surgery and cyclophotocoagulation. As the corneal diameter reflects disease severity, it can act as a rough guide to surgery choice. In general, corneal diameter less than 13 mm represents mild PCG, and surgical options include goniotomy or trabeculotomy. Corneal diameter between 13 and 16 mm indicates moderate severity, angle surgeries carry a higher chance of failure but may still be attempted. Other options include combined trabeculotomy-trabeculectomy, a traditional trabeculectomy or glaucoma drainage implant. Corneal diameter exceeding 16 mm indicates severe PCG, which cyclophotocoagulation may be the last resort.

As the manifestation of PCG tends to be more severe and occur in earlier years in non-Caucasians, below we will look into the literature on different surgical methods for PCG with a particular emphasis on non-Caucasian subjects.

**Table 3:** **Table 3:** Effect and side effects of antiglaucoma medications in PCG patients

*Class*		*Effect*		*Side effect*		*Nota bene*	
Beta blockers		Fair, no effect in 39-54% of patients		Possibility of apnea		To exclude asthma/cardiac anomaly before use. Check availability of 0.25% timolol, which is preferable in children	
Carbonic anhydrase inhibitors		Fair		Growth suppression, metabolic acidosis		―	
Prostaglandin analogs		Poor, no effect in 50-80% of patients		Mainly local side effects as observed in adults		Predictors of poor response include severity glaucoma and young age of presentation	
Alpha-2 agonists		―		CNS suppression		Contraindicated in children	
Parasympathomimetics		Poor		―		Limited use in pediatric patient	

### Goniotomy

Goniotomy reduces IOP by cutting into the abnormal trabecular meshwork, allowing the iris to drop posteriorly so as to deepen the angle recess. A clear cornea is a prerequisite. With proper case selection, goniotomy offers promising outcomes. It has been quoted that in 72% of PCG patients, one goniotomy was sufficient to reach a normal IOP and 88 to 94% patients would attain a normal IOP in two goniotomies.^36-38^ Predictors of higher success rate are those who present between 1 and 24 months after birth and lower degree of refractive error.^36,38^ The success rate in the western society is quite high, between 75 and 90%. In comparison, in South East Asian and the Middle East where the age of presentation is much earlier, the quoted success rate of goniotomy drops to approximately 50%. For PCG patients who present at birth to 1 month of age, the success rate after 1 to 2 goniotomies drops to 25%.^36^

In PCG patients with particular cloudy cornea, which prevents direct visualization of the angle structures, endoscopic goniotomy offers a allowing a direct view of irido-corneal angle and allows angle opening to 300°.^39-41^ The success rate of endoscopic goniotomy in PCG and secondary developmental glaucoma was quoted to be 50%.^42^

### Trabeculotomy

Trabeculotomy or goniotomy *ab externo,* was first described in 1960, lowering the IOP through inserting a trabeculotome into the Schlemm’s canal, which then tears through trabecular meshwork into the anterior chamber.^43^ Trabeculotomy is feasible in cases where the opaque cornea prevents good visualization of anterior chamber structures. It is also considered to be a more predictable and technically easier surgery. The procedure does not require surgical gonioscopy skills and is, thus, more similar to trabeculectomy, with a less steep learning curve.^44^ However, a direct comparison between goniotomy and trabeculotomy would suffer from selection bias, as trabeculotomy is often performed in more severe cases of glaucoma.

According to a retrospective study conducted in China, IOP less than or equal to 21 mm Hg could be achieved in 91 and 87% of patients 1 and 3 years respectively after trabeculotomy,^45^ which is comparable to Western data in the early 1980s of 75 to 90%.^46,47^

The 360° trabeculotomy is one modification of the surgery. Instead of opening approximating one-third of the chamber angle, the 360° trabeculotomy allows the entire angle to be opened in a single session. This is achieved by threading a 6-0 prolene suture or a lighted canaloplasty device through the Schlemm’s canal. A retrospective study comparing goniotomy and 360° trabeculotomy demonstrated that 92% PCG patients underwent 360° trabeculotomy has IOP controlled below 22 mm Hg with a single procedure, while only 57.5% of patients undergoing goniotomy has successful pressure control. The 360° trabeculotomy also achieves a longer duration of pressure control.^48^

Another modification to traditional trabeculotomy is the utilization of a modified probe tailored to the individual Schlemm’s canal curvature. The underlying principle is that patients with varying corneal diameter would have accordingly varying Schlemm canal curvature. Filous et al utilized one of three different probes in a retrospective study, stratifying patients according to their corneal diameters. This modified procedure was able to cause a mean decrease of 47% in IOP with surgical success achieved in 87% of the eyes.^49^ However, prospective study comparing traditional trabeculotomy with this modified trabeculotomy is not available.

### Combined Trabeculotomy-Trabeculectomy

In some centers, trabeculotomy is combined with trabeculectomy and is performed as a first line surgery in PCG patients, with trabeculectomy performed after trabeculotomy.

Mandal et al reported an IOP drop of 41.1% in PCG patients in India after the procedure, with IOP < 21 mm Hg maintained at 94.4 and 73% at the first and third year post-surgery respectively.^44^ Essuman et al demonstrated a success rate of 79% with combined trabeculotomy-trabeculectomy procedure in a western African population; however, only 44% patients maintained IOP < 21 mm Hg at 1 year of follow-up.^50^

As for whether combined trabeculotomy-trabeculectomy is superior to either trabeculotomy and trabeculectomy alone, Dietlein et al demonstrated a higher success rate of the combined procedure at 6 and 60 months of follow-up, but the survival analysis failed to demonstrate significant difference in the surgical outcome between the three procedures.^51^ A more recent study, on the other hand, demonstrated no significant difference in mean IOP lowering between groups with trabeculotomy and combined trabeculotomy-trabeculectomy, but statistically significantly higher success rate is achieved in combined surgery compared with trabeculectomy.^52^ One of the possible reason of higher success rate in the combined procedure may be due to the dual outflow pathway after the procedure.^53^

### Second Line Treatment

Following failed angle surgery, ophthalmologists either opt for a second angle surgery, or proceed with a filtration surgery, either a trabeculectomy or a drainage implant.

### Trabeculectomy

Primary trabeculectomy had been a popular procedure in the late 1980s and 1990s, with its success rate varying from 54 to 92.3% in PCG.^54,55^

In countries where surgical gonio lens are not readily available, primary trabeculectomy is still an effective and popular option. A retrospective study in Nigeria demonstrated statistically significant reduction of corneal haziness and reduction of IOP by 40%,^56^ while that in Egypt is 28.4%.^57^ In a comparison study conducted in China, trabeculectomy has a higher long-term success rate than trabeculotomy, with a less steep decline in the Kaplan-Meier survival analysis.^45^

Problems encountered in performing trabeculectomy in children are multifold: thick tenon, thin sclera, difficulty in identifying the limbus and an exuberant healing response. The success rate of trabeculectomy ranges from 35 to 50% at 1 year postoperation,^58^ Fulcher et al reviewed the long-term outcome for primary trabeculectomy in PCG, and found a lower success rate in children presenting before 1 year of age.^55^ In view of this, the use of antimetabolites has been gaining popularity.

### Antimetabolites

The use of mitomycin C (MMC) in children has been reported to improve success rates of surgery, quoted as 52 to 82%.^59,60^ Another study conducted in China yield success rate of 67.4% with a mean IOP drop of 31°%,^61^ which is comparable to the international data. As for the optimal concentration, a comparison study shows no statistically significant difference in IOP reduction, success rate or complication between use of 0.4 or 0.2 mg/ml concentration of topical MMC in refractory PCG patient.^62^

However, not all studies show a favorable outcome. A comparison study between primary trabeculectomy and augmented trabeculectomy with MMC shows no statistically significant difference in IOP lowering, but a higher complication rate with MMC use, including a higher rate of bleb-related endophthalmitis, ranging from 7 to 17%.^58,63^ As MMC often results in a thin avascular bleb, children are subject to more years of exposure to infection risk, thus resulting in a higher long-term infection rate overall.

Compared with MMC, the role of 5-fluorouracil (5FU) is limited in the pediatric population. Its use often requires additional postoperative injections, which requires additional general anesthesia sessions.^63^ Furthermore, 5FU is significantly less likely to achieve adequate IOP control when compared to MMC.^64^

In addition to trabeculectomy, some centers utilize 5FU as antimetabolite for combined trabeculotomy-trabeculectomy, with a success rate of 65%. However, it is noted that some patients may require up to 19 injections postoperatively, causing repeated risk of general anesthesia to the subject.^65^

### Glaucoma Draining Device (GDD)

Retrospective studies on Ahmed glaucoma valve (AGV) revealed a 28 to 49% reduction in the mean IOP and a success rate ranging from 63 to 97% at 1 year.^66-68^ The definition of success is similar across studies, which is defined as absence of serious complications or lost of light perception, with an acceptable IOP with or without the use of medication, thought the acceptable IOP ranges from 18 to 23 mm Hg across studies.^66-68^ Hispanic ethnicity and gender female have been reported to be independent predictors for failure of the GDD procedure, but the underlying reason has yet to be identified.^68^

Traditionally, insertion of GDD inevitably involves opening of a scleral flap, which may cause tube-migration related complications. A retrospective study conducted in Mexican children explored the outcomes of AGV implantation through a needle generated scleral tunnel, eliminating the need of a scleral flap. None of the patient had tube extrusion during the 6-month follow-up.^69^ Although the result is not statistically significant due to the small sampling size (CI < 1.5%), the study sheds insight to the alternative methods of GDD insertion.

With regards to the role of MMC application in GDD, a retrospective study studying 31 eyes revealed a success rate 2 years after AGV implantation if MMC was used intraoperatively, during the first 2 years of life (22.15 *vs* 16.25 months).^70^ It is speculated that MMC stimulates fibrosis along the AGV, leading to decreased success rate.

For studies comparing GDD with augmented trabecul ectomy, the majority are retrospective case series which show conflicting results. An age-matched study comparing augmented trabeculectomy with GDD in children under the age of two showed a significantly higher cumulative success rate in the GDD group at 6 years, with the success rate being 53% in the GDD group and 19% in the trabeculectomy group respectively.^71^ However, another study targeting older patients between 6 and 17 years of age demonstrated comparable total success rate in the two groups (88% in AGV and 86% in MMC-augmented trabeculectomy) and no statistically significant difference in IOP reduction. However, the patients receiving AGV are two times more likely to be on antiglaucoma medication for IOP control, and the AGV patients have a poorer visual outcome, with a visual acuity drop of three lines (28% *vs* 12%).^72^ Take note that the above two studies may have selection bias as secondary glaucoma were included, which are known for having a lower trabeculectomy success rate.

Pediatric GDD implantation requires extra considerations. The commonly used GDD include Baerveldt, Ahmed and Molteno implants with size varying from 96 to 250 mm^2^. The surface area of the implant appears roughly proportional to the pressure-lowering effect. In buphthalmic eyes, the use of adult-sized implants (e.g. AGV model FP7) may be considered. In small nanophthalmic eyes, the use of smaller implants (e.g. AGV model FP8) may be required.^73^ It has been suggested that GDD may be preferable to trabeculectomy when conjunctival scarring is evident,^74^ or in buphthalmic eyes with very thin sclera.^66^ It is hypothesized that as the bleb is located more posteriorly in GDD, the long-term risk of infection is, thus, theoretically lower.^66^ However, GDD is associated with more complications that requires re-operation than trabeculectomy (46% *vs* 13%), in particular displacement of tube leading to tube-endothelial touch and tube retraction.^68,71^

### Cyclodestructive Procedures

In general, cyclodestructive procedures are usually reserved until both angle procedures and another surgical modality has failed. The number of sessions required to achieve satisfactory IOP control is difficult to predict. Moreover, chronic inflammation together with relative aqueous under-secretion decreases the success rate of glaucoma drainage surgeries.

The traditional cyclocryotherapy yields a very low success rate of around 30%.^75,76^ This procedure is also associated with more postoperative inflammation, phthisical changes and is usually less preferable. Transscleral diode photocoagulation has a higher success rate, around 50% at 6 months, with a retreatment rate of 70% and requiring on average 2.2 ± 1.3 sessions. Endoscopic diode cyclodestruction has been described to deliver laser energy more precisely. The success rate after first procedure ranges from 17 to 34%, with mean IOP reduction of 23 to 30% after two sessions of procedure.^77,78^

Cyclodestructive procedure is known to be associated with multiple severe complications, including retinal detachment, hypotony, progression of vision loss and vitreous hemorrhage. Aphakic patients are in particular more prone to retinal detachment in endoscopic procedures^73,77,78^ Micropulse transscleral diode laser shows promising results with a seemingly better safety profile.^79,80^ Its application and effect in the pediatric population remains to be seen.

## CONCLUSION

Primary congenital glaucoma poses both a diagnostic and therapeutic challenge to the ophthalmologist. Advancement in research and technology has provided a clearer picture on how to tackle the disease, and we have tried to provide an update on PCG management with current evidence. However, due to the low incidence of PCG, studies in the field are usually retrospective, nonrandomized and have a limited sample size. Since the difficulty of management of PCG lies not only in the surgical technique but also in decision-making, clinicians should be aware of the study-design limitations.
